# Biological Characteristics and Bacterial Community of Invasive Pest *Corythucha ciliata* (Hemiptera: Tingidae)

**DOI:** 10.3390/insects16101055

**Published:** 2025-10-16

**Authors:** Tong-Pu Li, Bing-Ren Hao, Chen-Hao Wang, Jing-Jing Xu, Xiao-Tong Wang, Jia-Chu Xie, Zhi-Heng Wang, Shu-Cheng Ye, Lv-Quan Zhao

**Affiliations:** Co-Innovation Center for Sustainable Forestry in Southern China, College of Forestry and Grassland, Nanjing Forestry University, Nanjing 210037, China; tpli@njfu.edu.cn (T.-P.L.);

**Keywords:** *Corythucha ciliata*, invasive pest, biological characteristics, bacterial community, synergistic adaptation

## Abstract

The sycamore lace bug *Corythucha ciliata* (Hemiptera: Tingidae), an invasive North American forest pest, owes its strong dispersal and adaptability to biological characteristics and symbiotic microbes, but the underlying mechanisms have not been fully elucidated. This study examined its outdoor-collected (LYGO) and indoor-reared (LYGI) populations using morphological observation, biological parameter assessment, and *16S rRNA* sequencing. Key findings include: (1) Nymphs develop through five instars, with body size increasing significantly across stages; growth accelerated during 4th and 5th instars, reflecting a pattern of “low-instar accumulation and high-instar acceleration”. (2) Adult survival differed by sex, with females outliving males after 30 days; nymphs develop in 14.81 days, and each adult pair produced an average of 17 eggs, demonstrating a concentrated reproductive strategy; (3) Both populations shared dominant bacterial taxa (including the phyla Bacteroidota and Proteobacteria and the genus *Cardinium*) but diverged in non-dominant taxa; core microbial functions were conserved, while specific functions (e.g., glutathione S-transferase activity) varied. These results suggest a potential synergy between the insect’s biological characteristics (efficient development, concentrated reproduction) and the adaptive functions of its associated microbes in enhancing its invasiveness. The study supplements its basic biological data and offers a new view of its ecological adaptability.

## Simple Summary

The sycamore lace bug, an invasive insect originating from North America, threatens urban forests. Its strong ability to adapt to new environments is unclear, which limits effective control. This study compared the bug’s indoor-reared and outdoor-collected populations to explore how its biological characteristics and symbiotic microorganisms help it adapt. We found its young (called nymphs) go through 5 developmental stages, growing faster in the last two stages, adult females live longer than males after 30 days, and each adult pair produces approximately 17 eggs in a concentrated way. Both populations share key microorganisms, but non-key ones differ between indoor and outdoor groups to fit different environments. These biological characteristics and microorganisms may work together to boost the bug’s invasiveness. The study provides basic data on the bug and supports developing eco-friendly control methods (like targeting its key microorganisms), which could aid in protecting urban ecosystems.

## 1. Introduction

The sycamore lace bug *Corythucha ciliata* (Hemiptera: Tingidae) is an invasive forest pest that threatens urban garden ecosystems [1,2]. Both adults and nymphs damage host plants by piercing and sucking sap from sycamore leaves, causing white spots, wilting, and defoliation, which significantly impairs host plant photosynthesis and growth [3,4]. This pest is native to the mid-eastern region of North America and was first recorded in Changsha, China, in 2002 [5]. Owing to its strong dispersal ability, high environmental adaptability, and broad temperature tolerance, it has spread to multiple regions in China, including Hubei, Shanghai, Jiangsu, and Shandong, and continues to expand its range [5,6,7].

The damage caused by *C. ciliata* and its successful colonization are closely linked to its remarkable biological adaptability [8,9,10,11,12]. *C. ciliata* completes 2–5 generations per year, overwinters as adults, and exhibits strong cold hardiness [3,13,14,15,16]. However, despite growing concern regarding its invasive impact, systematic studies on its basic biological characteristics remain insufficient. For example, key parameters such as morphological differentiation markers of nymphal instars (e.g., stage-specific characteristics of body length, width, and surface area), sex differences in survival dynamics, and distribution patterns of fecundity have not been systematically quantified. Additionally, the association between symbiotic bacterial communities and the host’s biological characteristics or environmental adaptability remains to be clarified, especially due to a lack of in-depth analysis on how differences in microbial composition and function between indoor and outdoor populations support the host’s environmental adaptation. These research gaps limit a comprehensive understanding of *C. ciliata*’s invasion mechanisms.

Currently, chemical pesticides are the primary control measure for *C. ciliata*, supplemented by physical methods such as manual removal of infested leaves. However, long-term reliance on chemical control easily induces pesticide resistance and causes ecological pollution, which highlights the urgent need for sustainable biological control strategies [17,18]. Insect symbiotic microorganisms are key regulators of host ecological adaptation, playing critical roles in growth, development, nutrient metabolism, and stress resistance [19,20,21]. Their community composition and abundance are influenced by factors such as developmental stages and food resources, and the successful colonization of invasive pests is often closely associated with the plasticity of their symbiotic microorganisms [22,23,24,25]. Existing studies have shown that Tingidae insects harbor high microbial diversity, and their dominant phyla (e.g., Proteobacteria, Firmicutes) are involved in regulating the host’s nutrient metabolism and adaptation to host plants [26,27]. However, the association between symbiotic bacterial communities and the host’s biological characteristics or environmental adaptability in *C. ciliata*, a major invasive pest, remains poorly understood.

Therefore, this study focuses on biological characteristics and bacterial communities and those potential synergy effects in *C. ciliata*. By systematically measuring its growth and developmental indicators (body length, width, and surface area), survival dynamics, fecundity, and other biological parameters, and combining these measurements with comparative analysis of bacterial community diversity, composition, and function, we aim to reveal the potential role of symbiotic microorganisms in the host’s nutrient utilization and stress resistance, and infer their synergistic effects during *C. ciliata*’s invasion. The results will provide a scientific basis for understanding this pest’s ecological adaptability and dispersal mechanisms, and lay a theoretical foundation for developing green control technologies based on microbial regulation.

## 2. Materials and Methods

### 2.1. Collection and Rearing of Test Insects

To obtain experimental insect sources for morphological observation, biological characteristic research, and microbial analysis, adults, nymphs, and eggs of *C. ciliata* were collected from the undersides of sycamore leaves in the field in Lianyungang City, Jiangsu Province, in July 2024. These insects were brought back to the laboratory alongside the sycamore leaves to maintain the identical food source as in the field, and then reared under standardized conditions in an artificial climate chamber. The rearing conditions were set as follows: temperature at 26 °C ± 0.5 °C and relative humidity at 80% ± 5%. Rearing containers were large transparent plastic boxes, with the lid densely pierced with breathing holes using No. 3 or No. 4 insect needles. The bottom of the box was lined with 2 cm thick water-saturated sponges, and fresh sycamore leaves were laid flat on the sponges with their undersides facing up, onto which the lace bugs were transferred. Leaves were replaced and water was replenished every 7 days, with the water level maintained at 1/2 the height of the sponge. During replacement, adults and nymphs were transferred using a small brush, while dead individuals and excreta were removed to maintain a clean rearing environment.

### 2.2. Observation of Morphological Development of C. ciliata

To clarify the morphological characteristics and developmental dynamics of each nymphal instar of *C. ciliata*, four Petri dishes were selected, with 3 newly hatched nymphs placed in each dish for individual tracking. Breathing holes were pierced around the sidewall of each Petri dish, and wet filter paper was laid at the bottom. Sycamore leaves cut to an appropriate size were placed inside each dish, and the edges of the leaves (focusing on vein areas) were fixed with water-soaked absorbent cotton to keep them fresh. Four diagonally distributed fixed points were set in each dish, and water or leaves were replenished promptly to ensure sufficient food. The developmental status of nymphs was observed daily at the same time using a ZEISS M205FA microscope (ZEISS, Oberkochen, Germany), and the developmental duration was recorded until eclosion. By taking photos with a scale, body length, body width, and dorsal surface area of each nymph were measured. The average value of 3 nymphs in each Petri dish was taken as 1 biological replicate, with a total of 4 replicates.

### 2.3. Observation of Biological Characteristics of C. ciliata

The biological characteristics of *C. ciliata* were studied by determining its adult survival rate, developmental duration, and fecundity, respectively. Prior to these biological determinations, *C. ciliata* population was reared continuously for three generations in the laboratory to eliminate the potential impact of uncontrolled environmental factors on the insect.

#### 2.3.1. Adult Survival Rate

To explore the survival pattern of *C. ciliata* adults, newly emerged adults (15 males and 15 females) were selected and reared separately by sex (3–4 adults per Petri dish), with each dish numbered and marked. The same moisturizing and leaf replacement methods as in Section 2.2 were adopted. The survival and death of adults in each Petri dish were recorded daily at the same time until all 30 adults died, and the data were aggregated to calculate the daily survival rate.

#### 2.3.2. Developmental Duration

To clarify the complete life cycle (egg stage and each nymphal instar) of *C. ciliata*, newly emerged adults were paired (1 female and 1 male) in the same Petri dish (15 dishes in total). Breathing holes were pierced in the sidewall of each Petri dish, and round wet filter paper was laid at the bottom. Sycamore leaves cut to an appropriate size (preferring leaves with thick veins) were placed inside, and the edges of the leaves (focusing on vein areas) were fixed with water-soaked absorbent cotton to extend their freshness. Four diagonal fixed points were set in each dish. Mating and oviposition were observed daily at the same time under a stereomicroscope, and the oviposition time of black egg clusters at the junction of the main vein and lateral vein branches or under the main vein of the leaf was recorded. Observation continued until egg hatching, and the hatching time was recorded; the interval between the two was defined as the egg stage (days).

Sixteen newly hatched nymphs were selected (4 in each Petri dish, 4 dishes in total), and the same moisturizing and leaf replacement methods were adopted. Their growth and development were observed and photographed daily at the same time. The date was recorded when nymphs molted each time, and the exuviae were photographed until all nymphs eclosed. The interval between two molts was taken as the corresponding instar duration (days), where the 1st instar duration was defined as the time from hatching to the first molt.

#### 2.3.3. Fecundity

To evaluate the reproductive capacity of *C. ciliata*, newly emerged adults were paired (1 female and 1 male) in the same Petri dish (15 dishes in total, 15 males and 15 females, numbered 1–15) and reared with fresh sycamore leaves (leaves were freshly picked to extend freshness and avoid failure of egg hatching due to leaf withering). Mating and oviposition were observed daily at the same time under a stereomicroscope. After nymphs hatched, the number of hatched nymphs on that day was recorded and transferred to a new Petri dish using a brush. Until no new nymphs hatched for 3 consecutive days in the original Petri dish, the total number of hatched nymphs in each dish was counted, and the average value was calculated to reflect the fecundity per pair of adults.

### 2.4. Collection, Sequencing, and Data Assembly of Microbial Samples

To compare the differences in the internal bacterial communities between the outdoor-collected population (LYGO) and the indoor-reared population (LYGI), microbial diversity determination was performed on the two populations. Each population had 3 replicates, with 5 adults in each group, totaling 6 samples. After sample collection, the body surface was cleaned with 75% ethanol for 90 s to remove attached microorganisms, followed by 3 rinses with sterile deionized water to thoroughly eliminate residual ethanol and contaminants. Due to the small body size and difficulty in dissecting *C. ciliata*, as well as to maximize the reflection of the total bacterial community in its body, the collected samples were directly used for DNA extraction and sequencing.

Genomic DNA of samples was extracted using the Qiagen DNeasy^®^ Blood & Tissue Kit (Qiagen, Düsseldorf, Germany). The V3–V4 hypervariable regions of the bacterial *16S rRNA* gene were amplified by PCR using specific primers (forward primer: 5′-CCTAYGGGRBGCASCAG-3′; reverse primer: 5′-GGACTACNNGGGTATCTAAT-3′) [28]. PCR products were separated by 2% agarose gel electrophoresis and then purified using the AxyPrep DNA Gel Extraction Kit (Axygen, Union City, CA, USA). After quantification with Qubit 3.0, a PE250 (2 × 250 bp paired-end) sequencing library was constructed, and high-throughput sequencing was performed on the Illumina NovaSeq 6000 platform (Illumina, San Diego, CA, USA). Raw data were distinguished by index sequences and saved in FASTQ format. Using DADA2 (a QIIME 2 plugin), sequence quality filtering (removal of sequences with length < 200 bp or average quality score < 20), denoising, and chimera removal were performed to directly generate amplicon sequence variants (ASVs); singleton ASVs were further removed to reduce noise. The optimized ASV representative sequences were compared with the Silva database to generate an ASV table (Table S1).

### 2.5. Analysis of Microbial Diversity and Function

To reveal the differences in bacterial community structure, diversity, and function between outdoor and indoor populations, alpha diversity analysis was used to calculate species richness (observed ASV number, Chao1 index, ACE index) and evenness (Shannon index, Simpson index), which were used to describe the bacterial community diversity of different populations [26]; Chao1 and Shannon indices were selected for visual display. For beta diversity analysis, permutational multivariate analysis of variance (PERMANOVA) was used to test differences in bacterial communities between populations, and principal coordinate analysis (PCoA) based on Bray–Curtis distance (implemented in QIIME 2) was used for visualization. In addition, community structure diagrams were drawn based on the relative abundance of ASVs at the phylum and genus levels (genera with relative abundance ranking outside the top 15 were merged into “others” for simplified display).

### 2.6. Data Processing and Statistics

To ensure the statistical reliability of experimental data, the normality and homoscedasticity of data on body length, body width, and surface area were tested using the Shapiro–Wilk test and Levene test. Data meeting the conditions were compared pairwise using one-way analysis of variance (one-way ANOVA) combined with the post hoc least significant difference (LSD) method; data not meeting the conditions were compared pairwise using the Kruskal–Wallis test combined with the post hoc Dunn’s method. The independent sample Student’s *t*-test was used to compare the lifespan of lace bugs, bacterial diversity, the relative abundance of microorganisms, and the abundance of KEGG functional annotations. A *p*-value < 0.05 was considered statistically significant in all statistical analyses.

## 3. Results

### 3.1. Morphological Characteristics and Growth Quantitative Analysis of C. ciliata at Different Developmental Stages

**Figure 1:**
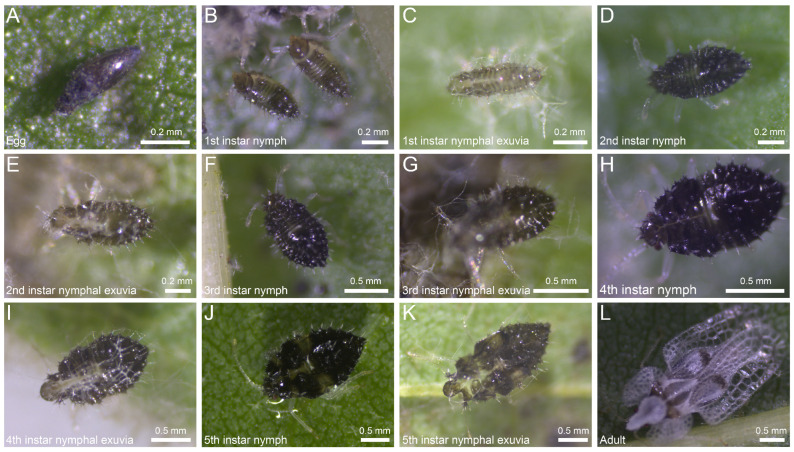
Morphological characteristics of *C*. *ciliata* throughout its complete developmental stages. The images, respectively, demonstrate the morphological features of eggs, nymphs, exuviae (1st–5th instars), and adults of *C. ciliata*.

To elucidate the patterns of morphological differentiation during the growth and development of *C. ciliata*, continuous observation and quantitative measurements were conducted, clarifying stage-specific markers and growth dynamics. The results showed that eggs were eggplant-shaped, with a black oval egg cap at the top, and females mostly laid eggs in clusters at the junction of the main vein and branch veins on the underside of leaves (Figure 1A). Nymphs were wingless and had a flat elliptical body (5th instar) (Figure 1B–K). Newly hatched 1st instar nymphs had transparent bodies, which later turned dark brown. The pronotum of 1st–2nd instar nymphs was narrower than the abdomen, with a light and transparent body color. The pronotum of 3rd–5th instar nymphs gradually became as wide as the abdomen; 3rd–4th instar nymphs had a shiny black body color, and 5th instar nymphs were broadly fusiform with brown markings on the thorax. Adults were macropterous, with a conical head hood on the head, piercing-sucking mouthparts, a white dorsal surface, forewings exceeding the end of the abdomen with reticulated transparent edges, 4 black spots on the back, forewings lying flat in a rectangular shape when at rest, yellowish antennae and legs, and dark red compound eyes (Figure 1L).

**Figure 2:**
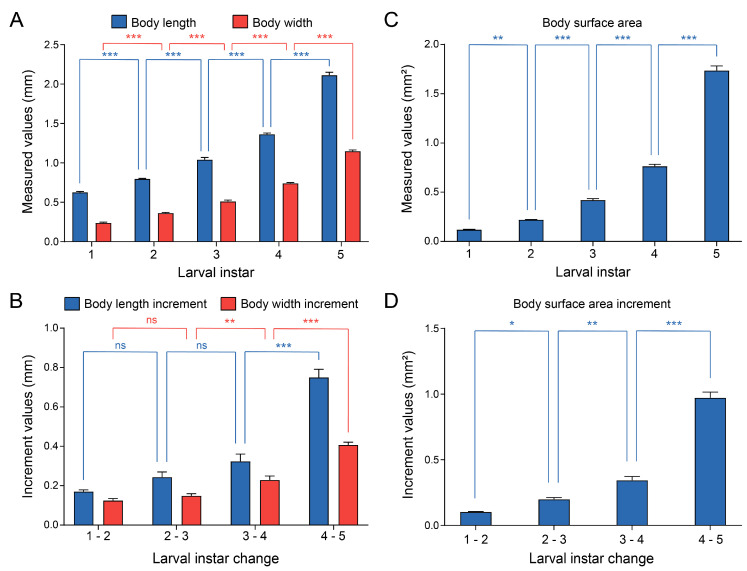
Growth profiles of *C. ciliata* larvae. (**A**) Measured body length (blue) and body width (red) across 1st–5th instars larvae. (**B**) Incremental changes in body length (blue) and body width (red) between consecutive larval instars (1–2, 2–3, 3–4, 4–5). (**C**) Measured body surface area across 1st–5th instars larvae. (**D**) Incremental changes in body surface area between consecutive larval instars (1–2, 2–3, 3–4, 4–5). Statistical significance is denoted as: * *p* < 0.05, ** *p* < 0.01, *** *p* < 0.001, and ns (non-significant) (*n* = 4).

The changes in morphological characteristics during the growth and development of *C. ciliata* were quantitatively analyzed. The results showed that both body length and body width of nymphs increased significantly with instar progression, with extremely significant differences between adjacent instars (one-way ANOVA with post hoc LSD test, *p* < 0.001) (Figure 2A). The incremental changes in body length and width between consecutive instars were further statistically analyzed: there were no significant differences in the increments of body length and width during the transition from 1st to 2nd instar and from 2nd to 3rd instar; however, significant differences in increments were observed during the transition from 3rd to 4th instar and from 4th to 5th instar, indicating that the growth and development of *C. ciliata* nymphs accelerated gradually with instar progression (Figure 2B). The body surface area increased continuously and significantly from the 1st to 5th instar: there was a highly significant difference between the 1st and 2nd instars (one-way ANOVA with post hoc LSD test, *p* < 0.001), and extremely significant differences were observed in subsequent instar transitions (one-way ANOVA with post hoc LSD test, *p* < 0.001), reflecting the continuous expansion of body surface area (Figure 2C). In terms of body surface area increment: the increment was significant during the transition from 1st to 2nd instar (one-way ANOVA with post hoc LSD test, *p* < 0.05), highly significant during the transition from 2nd to 3rd instar (one-way ANOVA with post hoc LSD test, *p* < 0.01), and extremely significant in subsequent instar transitions (one-way ANOVA with post hoc LSD test, *p* < 0.001), with the most significant growth occurring during the transition from 4th to 5th instar (Figure 2D). These results indicated that the body length, body width, and body surface area of *C. ciliata* nymphs all increased with instar progression, and the growth increments were more prominent in older nymphs (4th–5th instars).

### 3.2. Survival Dynamics, Developmental Duration, and Reproductive Characteristics of C. ciliata

**Figure 3:**
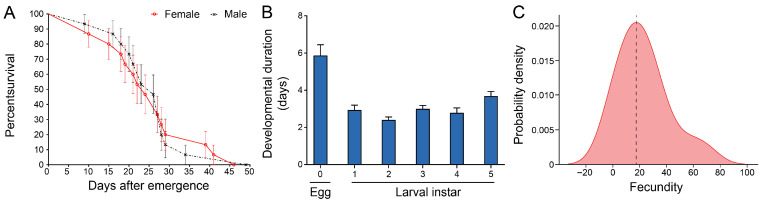
Biological characteristics of *C. ciliata*. (**A**) Survival curves of female and male adult of *C. ciliata* (*n* = 60). (**B**) Developmental duration for eggs and 1st–5th instar larvae of *C. ciliata* (*n* = 15). (**C**) Probability density distribution of fecundity in *C. ciliata* (*n* = 15).

To analyze the biological basis of population expansion of *C. ciliata*, this study analyzed the characteristics of adult survival, nymphal development, and fecundity. The initial survival rate of newly emerged male and female adults was nearly 100%. The male survival rate was slightly higher in the first 20 days; a death peak occurred between 25–30 days, with a significant decrease in survival rate and narrowed differences between males and females. After 30 days, the female survival rate surpassed that of males and persisted until population extinction (maximum lifespan < 50 days). Statistical analysis confirmed no significant difference in lifespan between male and female adults, revealing the stage differentiation and gender balance characteristics of survival dynamics (Figure 3A). In terms of developmental duration, the egg stage was the longest (5.87 days). Among 1st–5th instar nymphs, the 2nd instar had the shortest duration (2.4 days), and the 5th instar duration was significantly prolonged (3.69 days), with a total nymphal developmental duration of 14.81 days. This showed a rhythm of “egg stage as foundation, rapid development in middle instars, and preparation for eclosion in the final instar,” which corresponded to the stages of adult survival dynamics (Figure 3B). In terms of fecundity, females laid eggs intermittently at leaf veins after mating, with scattered egg clusters. The probability density distribution of fecundity showed a unimodal characteristic (peak median: 17 eggs), reflecting the centralization law of fecundity. Combined with the reproductive window in the late adult survival period and the efficient developmental process of nymphs, this further supported the population expansion potential of *C. ciliata* (Figure 3C). In summary, *C. ciliata* constructs a synergistic biological strategy through stage adaptation of survival dynamics, efficiency optimization of developmental rhythm, and concentrated output of fecundity, laying a core foundation for population expansion.

### 3.3. Comparison of Bacterial Communities Between Indoor and Outdoor Populations

**Figure 4:**
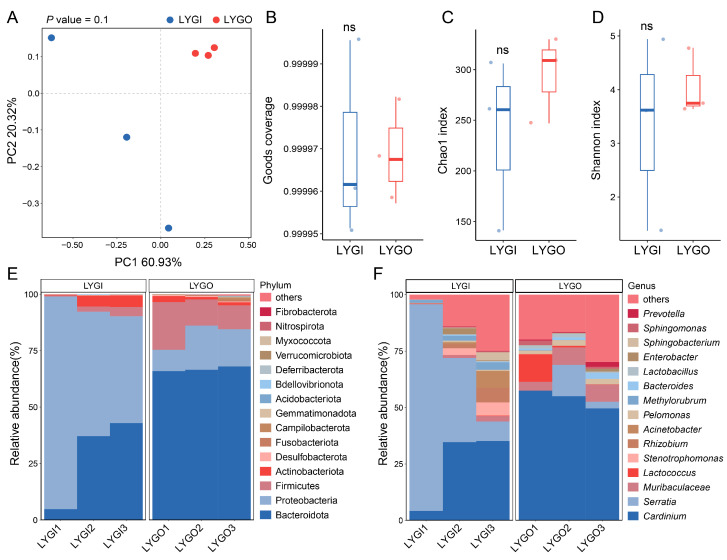
Bacterial community diversity and compositional profiles in *C. ciliata*. (**A**) Principal coordinate analysis (PCoA) illustrating beta-diversity patterns between LYGI and LYGO groups (*n* = 3). The horizontal axis represents principal coordinate 1 (PC1, explaining 60.93% of the total bacterial community variance), and the vertical axis represents principal coordinate 2 (PC2, explaining 20.32% of the total bacterial community variance). (**B**–**D**) Box plots depicting Good’s coverage (**B**), Chao1 index ((**C**), community richness), and Shannon index ((**D**), species evenness) of the bacterial communities in LYGI and LYGO groups (ns = non-significant). (**E**,**F**) Taxonomic distribution of the bacterial communities at the phylum level (**E**) and genus level (**F**) comparing LYGI and LYGO groups. Only the top 15 taxa by relative abundance are displayed, while the remaining taxa are grouped as “others”. Abbreviations: LYGI, indoor-reared *C. ciliata* population; LYGO, outdoor-collected *C. ciliata* population.

To analyze the population differences in the internal bacterial communities of *C. ciliata*, the bacterial community diversity and composition characteristics of the indoor-reared population (LYGI) and outdoor-collected population (LYGO) in Lianyungang were compared. Principal coordinate analysis (PCoA) showed a tendency of separation in bacterial communities between LYGI and LYGO, with PC1 and PC2 explaining 60.93% and 20.32% of community variation, respectively (Figure 4A). Alpha diversity analysis indicated no significant differences (ns) between the two groups in sequencing completeness (Good’s coverage, Figure 4B), community richness (Chao1 index, Figure 4C), and species evenness (Shannon index, Figure 4D). In terms of taxonomic composition, at the phylum level, Bacteroidota was the dominant phylum in both LYGI and LYGO, followed by Proteobacteria; the relative abundances of non-dominant phyla such as Actinobacteriota and Desulfobacterota differed between the two groups (Figure 4E). At the genus level, *Cardinium* was the dominant genus common to both groups, and the relative abundances of genera such as *Prevotella*, *Sphingomonas*, and *Serratia* showed obvious differentiation between LYGI and LYGO (Figure 4F).

### 3.4. Comparison of Bacterial community Functions Between Indoor and Outdoor Populations

To evaluate the functional potential of the internal bacterial community of *C. ciliata* and its response pattern to the environment, and to analyze the association between microbial functions and host adaptability, this study compared the microbial functional differences between indoor and outdoor populations (Table 1). The results showed no significant differences in major microbial functions between the two populations, as reflected by the top 10 KEGG Orthologs (KO) in terms of abundance, which showed no significant differences between the two groups. These functions mainly involved material transport (e.g., iron complex outer membrane receptor protein, ABC transport system-related proteins), transcriptional regulation (e.g., RNA polymerase σ-70 factor, LacI family transcriptional regulator), and stress response (e.g., cold shock protein), indicating that the high-abundance microbial functions of the two groups were generally convergent.

In addition, the two populations differed in some microbial functions. Among the top five functions with significant differences in abundance: oxygen-independent coproporphyrinogen III oxidase, N-acetylmuramoyl-L-alanine amidase, *23S rRNA* pseudouridine synthase, and CAAX protease family protein were significantly more abundant in LYGO than in LYGI (Student’s *t*-test, *p* < 0.05); only glutathione S-transferase (a detoxification-related enzyme) was extremely significantly more abundant in LYGI than in LYGO (Student’s *t*-test, *p* < 0.01). These results indicated that indoor and outdoor environments had no significant impact on the major microbial functions of *C. ciliata*, but may have selective effects on functions related to porphyrin metabolism, cell wall degradation, ribosome modification, and detoxification.

## 4. Discussion

This study systematically investigated the morphological development, biological characteristics, and dynamics of symbiotic bacterial communities of the invasive pest *C. ciliata*, revealing three key findings: (1) The nymphs of this pest undergo 5 instars with significant morphological differentiation; body length, width, and surface area increase significantly with instar progression, with accelerated growth in the 4th–5th instars; (2) Adult survival dynamics exhibit sexual differences, with female survival rates surpassing males after 30 days; the total nymphal developmental duration is 14.81 days, and each pair of adults (1 female and 1 male) produces a peak median of 17 eggs, showing a concentrated reproductive pattern; (3) Regarding bacterial communities, indoor and outdoor populations share dominant phyla (Bacteroidota, Proteobacteria) and genus (*Cardinium*), but differ in non-dominant taxa (e.g., *Prevotella*, *Sphingomonas*, *Serratia*); core microbial functions (e.g., material transport, stress response) are convergent, while specific functions (e.g., glutathione S-transferase) differ significantly. These findings construct a “morphology-physiology-microbiology” three-dimensional evidence chain, providing a new perspective for explaining the strong invasiveness of *C. ciliata*.

Regional adaptability and strategic optimization of biological characteristics form the basis of *C. ciliata*’s successful invasion. This study found that the egg stage of the Lianyungang population (5.87 days) is shorter than that of the Hubei population (9.34 days), but the nymphal duration (14.81 days) is longer than that of the Hubei population (10.23 days) [29,30]. This suggests a potential increase in annual generation number and a prolonged nymphal damage period, reflecting differences in environmental adaptation among geographic populations [31]. Nymphal development exhibits the characteristic of “low-instar accumulation and high-instar acceleration”: instars 1–3 show slow growth, which may serve as a nutritional reserve stage [32]; instars 4–5 display significantly increased increments in body length, width, and surface area, which can shorten the generation cycle to cope with environmental pressures [33]. In adult survival dynamics, the death peak between 25–30 days, combined with the late-stage female survival advantage and the concentrated reproductive pattern (with a peak median of 17 eggs), aligns with the efficient replenishment characteristic of the “r-strategy” in invasive species [34]. Additionally, the synergistic effect between adult cold hardiness and the upregulated expression of host heat shock protein-related genes after low-temperature induction, coupled with wind dispersal capacity, collectively enhances the geographic expansion potential of *C. ciliata* [14,15].

Environmental plasticity in bacterial communities provide crucial support for host adaptation. Although indoor and outdoor populations share core microbial groups (with Bacteroidota as the most abundant phylum and *Cardinium* as the dominant genus), significant differences exist at the genus level: the indoor population harbors both *Serratia* and *Cardinium*, while the outdoor population is dominated exclusively by *Cardinium* (i.e., the relative abundance of *Serratia* in the outdoor population is lower than that in the indoor population). This reflects the shaping effect of rearing environments (constant temperature and humidity) on microbial community structure [27,35,36]. Alpha diversity analysis shows a decreasing trend in richness and diversity of indoor populations, which may be related to environmental simplicity and limited food resources [37,38]; in contrast, the higher diversity in field populations may arise from rich microbial sources in complex natural environments [27,39]. Functionally, the enrichment of porphyrin metabolism and cell wall degradation functions in field populations may enhance the ability of *C. ciliata* to utilize complex nutritional conditions in the wild [40]; the high abundance of glutathione S-transferase (a detoxification-related enzyme) in indoor populations is associated with detoxification needs in stable rearing environments, supporting the hypothesis that “microbial functions are specifically optimized with the environment” [9].

Synergistic adaptation between biological characteristics and bacterial communities is the key mechanism underlying the successful invasion of *C. ciliata*. The rapid growth of late-instar nymphs may rely on conserved microbial material transport functions (e.g., ABC transporters) to provide efficient energy supply for growth [41,42]; adult cold hardiness and prolonged survival may be related to microbial stress response genes (e.g., cold shock proteins), while concentrated reproduction may benefit from energy allocation regulation mediated by *Cardinium* [43,44]. Previous studies have shown that more significant synergies are manifested in environmental stress responses: the abundance of Acetobacter in field populations increases 5-fold at 35 °C, and this bacterium may interact with the host’s heat shock response by degrading heat stress toxins [17]. This “host phenotype-microbial function” bidirectional regulation enhances the adaptive flexibility of *C. ciliata* to heterogeneous environments.

## 5. Conclusions

In summary, this study clarifies that *C. ciliata* achieves successful invasion through a potential synergy involving “morphological differentiation rules–biological strategy optimization–bacterial community plasticity”. These findings not only supplement basic biological data for this species but also provide references for green pest control, such as microbial intervention targeting core symbiotic bacteria (e.g., *Cardinium*) and functional pathways (e.g., stress response genes). Future research should further quantify the contribution weight of microbial functional genes to host adaptation and verify the field effectiveness of “insect–microbe” synergistic regulation technologies, thereby providing scientific support for ecosystem protection.

## Figures and Tables

**Table 1:** Comparison of microbial functions between LYGI and LYGO populations of *C. ciliata*.

KEGG		KO Abundance (Mean ± SD)		*p* Value
Pathway	Description	LYGI	LYGO	Significant
K03088	RNA polymerase sigma-70 factor, ECF subfamily	342,973.21 ± 172,149.27	549,280.53 ± 8361.1	ns
K02014	iron complex outermembrane recepter protein	344,525.58 ± 61,795.22	415,048.68 ± 15,882.57	ns
K02004	putative ABC transport system permease protein	176,857.04 ± 111,678.23	358,744.23 ± 32,259.44	ns
K01992	ABC-2 type transport system permease protein	205,153.58 ± 49,720.21	300,326.43 ± 33,022.48	ns
K01990	ABC-2 type transport system ATP-binding protein	158,257.43 ± 75,933.69	303,297.21 ± 49,860.48	ns
K02529	LacI family transcriptional regulator, galactose operon repressor	216,059.18 ± 6935.44	222,089.19 ± 18,885.97	ns
K06147	ATP-binding cassette, subfamily B, bacterial	143,892.3 ± 87,322.22	274,952.44 ± 23,509.43	ns
K00059	3-oxoacyl-[acyl-carrier protein] reductase	211,219 ± 27,538.19	202,613.6 ± 13,717.21	ns
K02003	putative ABC transport system ATP-binding protein	131,352.23 ± 77,379.26	272,946.78 ± 36,340.53	ns
K03704	cold shock protein	178,252.38 ± 23,936.65	142,347.26 ± 25,298.1	ns
K02495	oxygen-independent coproporphyrinogen III oxidase	96,967.53 ± 10,248.56	121,149.63 ± 4397.21	*
K00799	glutathione S-transferase	151,199.27 ± 20,088.62	56,047.93 ± 6299.75	**
K01448	N-acetylmuramoyl-L-alanine amidase	88,364.23 ± 8408.03	116,465.17 ± 7513.13	*
K06180	23S rRNA pseudouridine1911/1915/1917 synthase	67,500.12 ± 28,706.83	132,536.65 ± 11,187.28	*
K07052	CAAX protease family protein	75,988.43 ± 14,811.74	119,833.64 ± 16,072.32	*

The top 10 microbial functions by abundance and the top 5 microbial functions with significant differences in abundance are displayed. Data are presented as mean ± SD. Statistical significance is denoted as: * *p* < 0.05, ** *p* < 0.01, and ns (non-significant) (*n* = 4). Abbreviations: LYGI, indoor-reared *C. ciliata* population; LYGO, outdoor-collected *C. ciliata* population.

## Data Availability

The raw data supporting the conclusions of this article will be made available by the authors on request. The *16S rRNA* amplicon sequencing data used in this study has been successfully uploaded to the NCBI SRA database with the corresponding BioProject accession number: PRJNA1313396. You can use this accession number to access the data through the NCBI database.

## Supplementary Materials

The following supporting information can be downloaded at: https://www.mdpi.com/article/10.3390/insects16101055/s1, Table S1: Taxonomic characteristics and summary of representative sequences for Amplicon Sequence Variants.

## Abbreviations

The following abbreviations are used in this manuscript:

LYGI	Indoor-reared *C. ciliata* population
LYGO	Outdoor-collected *C. ciliata* population
